# Delayed-onset chronic mandibular osteomyelitis following Lemierre’s syndrome: a case report

**DOI:** 10.1186/s40902-026-00504-0

**Published:** 2026-04-02

**Authors:** Min-Seo Kim, Young-Hee Kim, Sang-Yoon Park, Hyung-Chan Lee, Tae-Yoon Park, Byoung-Eun Yang, Soo-Hwan Byun

**Affiliations:** 1 Department of Oral and Maxillofacial Surgery, Hallym University Sacred Heart Hospital, Anyang, Republic of Korea; 2 Department of Oral and Maxillofacial Radiology, Hallym University Sacred Heart Hostpital , Anyang, Republic of Korea; 3 Graduate School of Clinical Dentistry, Hallym University, Chuncheon, Republic of Korea; 4 Institute of Clinical Dentistry, Hallym University, Chuncheon, Republic of Korea; 5 Dental AI-Robotics Center, Hallym University Sacred Heart Hospital, Anyang, Republic of Korea

**Keywords:** Lemierre’s syndrome, Mandibular osteomyelitis, External jugular vein, Facial vein

## Abstract

**Background:**

Lemierre’s syndrome (LS) is a rare condition in the dental field but represents a potentially fatal complication of odontogenic infection. Although osteomyelitis has been reported in association with LS, delayed-onset chronic mandibular osteomyelitis occurring after completion of LS treatment is exceedingly rare.

**Case presentation:**

A 16-year-old previously healthy male developed LS following odontogenic infection, complicated by septic thrombophlebitis of the external jugular and facial veins and septic pulmonary emboli. After surgical drainage and antibiotic therapy, clinical recovery was achieved. However, during post-treatment follow-up, radiographic evaluation revealed chronic osteomyelitis involving the entire mandible including both condyles. Extended antimicrobial therapy and close radiologic surveillance resulted in stabilization of the osteolytic changes and functional recovery.

**Conclusion:**

This case highlights a rare presentation of delayed-onset mandibular osteomyelitis as a sequela of Lemierre’s syndrome. Long-term follow-up after apparent resolution of LS is essential, particularly in odontogenic cases, and dental practitioners should be aware of this uncommon but clinically significant complication.

**Supplementary Information:**

The online version contains supplementary material available at 10.1186/s40902-026-00504-0.

## Background

Lemierre’s syndrome (LS) was systematically characterized by the French bacteriologist André Lemierre in 1936. LS is defined as septic thrombophlebitis of the internal jugular vein (IJV) with metastatic septic emboli, most commonly arising from head and neck infections caused by *Fusobacterium necrophorum* [1]. Before the advent of antibiotics, LS carried a mortality rate approaching 90%. With widespread antibiotic use, both incidence and mortality markedly declined, and the syndrome became known as the “forgotten disease” [2]. However, recent factors—including decreased prescriptions of oral antibiotics, rising antimicrobial resistance, and advances in imaging diagnostics—have contributed to a resurgence of reported cases, with mortality still ranging from 4% to 18% [3].

The syndrome typically occurs in otherwise healthy young adults between 16 and 25 years of age, often preceded by pharyngitis or tonsillitis [4]. Because its initial manifestations are nonspecific, diagnosis is frequently delayed, which can result in treatment delays and increased mortality [5]. Imaging modalities are indispensable for timely diagnosis. Computed tomography (CT), ultrasonography, and magnetic resonance imaging (MRI) are commonly employed to assess thrombus formation in the IJV and metastatic lesions, with contrast-enhanced CT regarded as the most reliable diagnostic tool [6, 7].

Osteomyelitis is a recognized complication that may accompany LS, and although involvement of the mandible is rare, it is not unprecedented [8, 9]. When LS of odontogenic origin – which accounts for approximately 2% of cases – is accompanied by mandibular osteomyelitis, the overlapping clinical features may hinder dental practitioners from making a prompt diagnosis and providing appropriate management [10].

In this case report, we present a case developed chronic mandibular osteomyelitis during post treatment follow-up rather than during active treatment for LS. Prolonged antibiotic therapy, combined with continuous radiographic monitoring, resulted in stabilization of the osteolytic process, as confirmed by panoramic radiography and CT. This report aims to underscore the importance of early recognition, accurate diagnosis, appropriate management, and meticulous long-term follow-up of LS.

## Case presentation

A 16-year-old previously healthy male presented to the emergency department with acute onset of neck pain, swelling, and high fever. Two weeks earlier, he had undergone extraction of the left mandibular second molar and pulpotomy of the right mandibular second molar. Following these procedures, he developed progressively worsening dysphagia, cervical swelling, and pain, subsequently accompanied by trismus and purulent intraoral discharge one week prior to admission. At presentation, his body temperature was 39.4 °C, and physical examination revealed bilateral swelling and tenderness of the buccal, mandibular, and submandibular regions. Maximum mouth opening was restricted to 1.5 finger breadths.

**Fig. 1:**
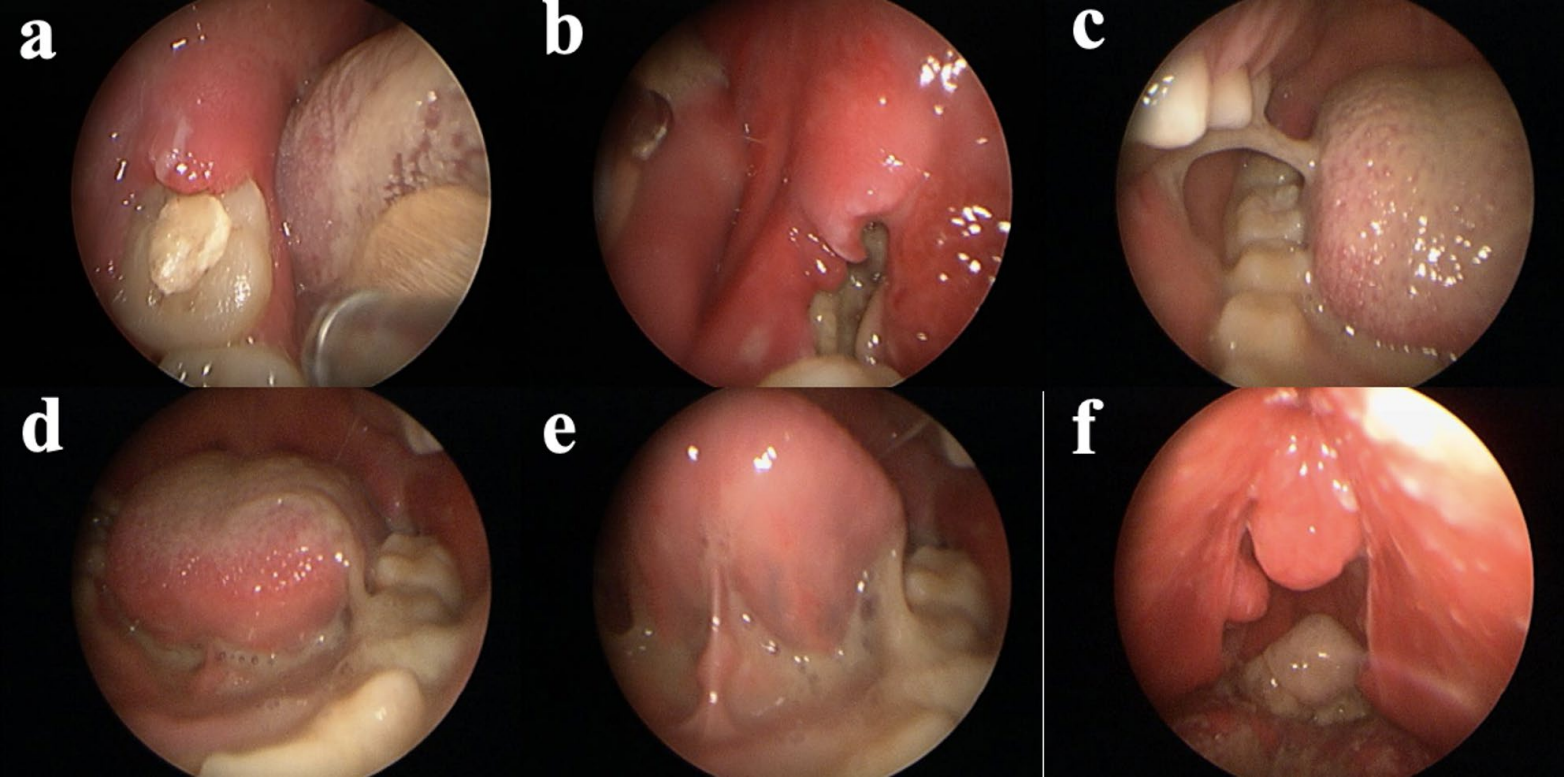
Laryngoscopy. **a** Distal gingival swelling around the right mandibular second molar. **b** purulent drainage from the extraction socket. **c** purulent exudate involving right buccal mucosa. **d**, **e** mouth floor. **f** oropharynx

**Fig. 2:**
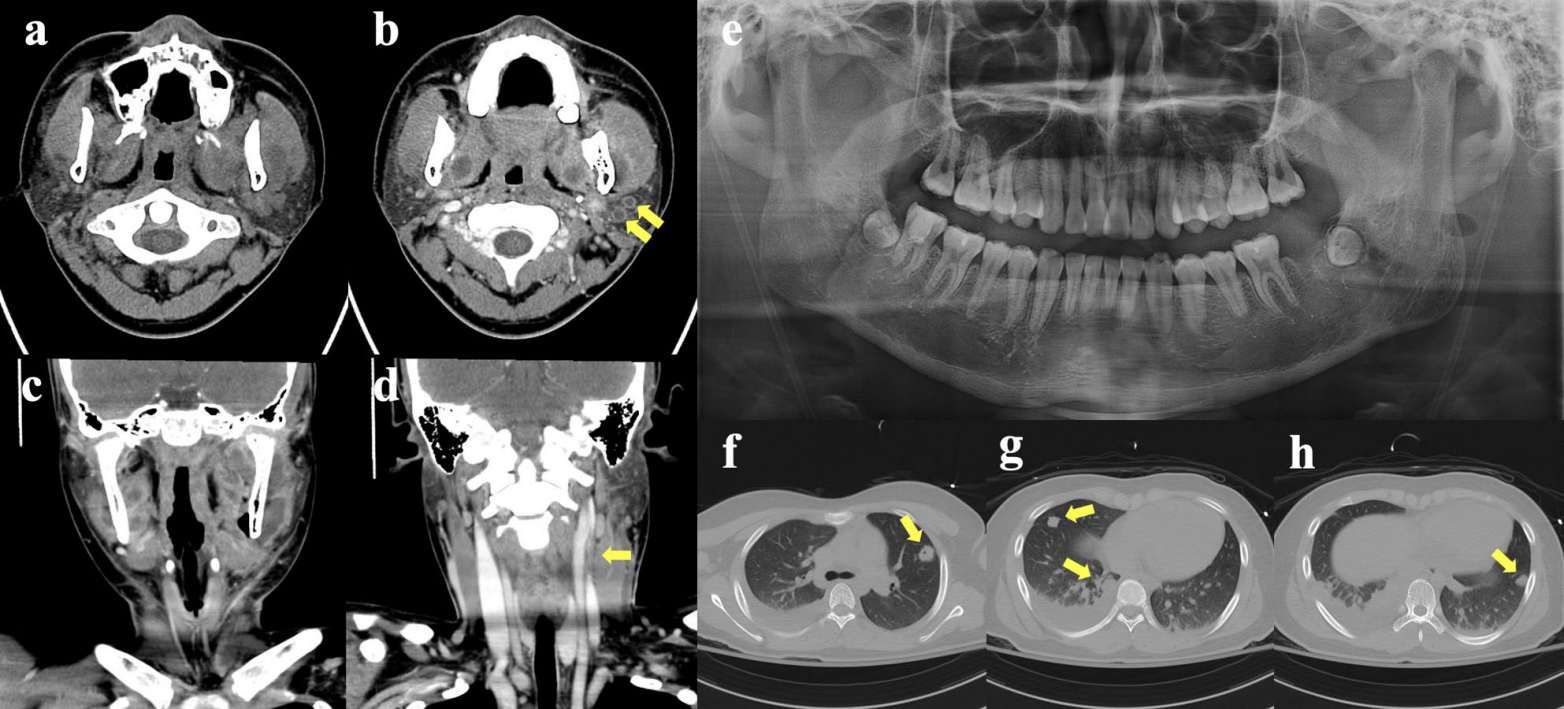
Initial assessment. **a**,** b**, **c**,** d** Contrast enhanced neck CT; multifocal abscess and myositis in both masticator spaces and masseter muscle. (arrows: acute thrombophlebitis in left EJV and facial vein) **e** Panoramic radiograph. **f**,** g**,**h** Chest CT; right pleural effusion. (arrows: septic emboli in both lung)

Intraoral examination demonstrated distal gingival swelling around the right mandibular second molar (Fig. 1a) and purulent drainage from the extraction socket of the left mandibular second molar (Fig. 1b). Laryngoscopic evaluation revealed extensive purulent exudate involving the right buccal mucosa (Fig. 1c), floor of the mouth (Fig. 1d, e), and oropharynx (Fig. 1f). A panoramic radiograph showed no additional odontogenic or osseous abnormalities (Fig. 2e). Contrast-enhanced neck computed tomography revealed

1. multiple abscesses and myositis in the bilateral masseteric and masticator spaces;
2. acute thrombophlebitis of the left external jugular vein (EJV) and facial vein; and
3. cellulitis involving the left oropharyngeal wall and submandibular space (Fig. 2a, b,c, d).

These findings were suggestive of necrotizing fasciitis with acute septic thrombophlebitis. Chest radiography demonstrated blunting of the right costophrenic angle, and chest CT confirmed right-sided pleural effusion and multiple septic emboli in both lungs (Fig. 2f, g,h).

Laboratory evaluation revealed marked leukocytosis and elevation of inflammatory markers including ESR, CRP, amylase, and procalcitonin (Table 1). Blood cultures identified *Fusobacterium necrophorum* as the causative pathogen.

**Table 1:** Laboratory results of the patient on admission

	**Initial value**	**Reference**		**Initial value**	**Reference**
WBC (/µL)	35,600	4500~10,000	CK (IU/L)	538	22~269
Hb (g/dL)	14.6	14 ~ 17	LD (IU/L)	457	0 ~ 249
Platelet (x$\:{10}^{3}$/µL)	147	130 ~ 450	Amylase (U/L)	220	28 ~ 100
Neutrophil (%)	96.0	40 ~ 74	BUN (mg/dL)	22.8	5.0 ~ 23.0
Lymphocyte (%)	3	0 ~ 9	Cr (mg/dL)	0.94	0.7 ~ 1.2
ESR (mm/hr)	39	0 ~ 15	CRP (mg/dL)	299.45	0.0 ~ 5.0
AST (IU/L)	300	8 ~ 38	Procalcitonin (ng/mL)	86.80	0 ~ 0.06
ALT (IU/L)	158	5 ~ 43	PT INR	1.34	0.88 ~ 1.13
Total bilirubin (mg/dL)	1.13	0.2 ~ 1.2	aPTT (sec)	35.1	29.1 ~ 45.1

The patient was admitted to the Department of Oral and Maxillofacial Surgery and underwent incision and drainage under general anesthesia, along with extraction of the right mandibular second molar. Two intraoral and two extraoral drains were placed. On hospital day 3, he was transferred to the Department of Pediatrics for systemic management and was diagnosed with LS. Broad-spectrum antimicrobial therapy with piperacillin/tazobactam, meropenem, and metronidazole was initiated. Despite therapy, high fever persisted, and on hospital day 21, chest radiography revealed new-onset right-sided empyema. Chest CT demonstrated pleural thickening and an air–fluid level within the pleural cavity, prompting transfer to the Department of Thoracic Surgery. Video-assisted thoracoscopic surgery (VATS) was performed on hospital day 27. The postoperative course was favorable, and the patient was discharged on hospital day 30.

**Fig. 3:**
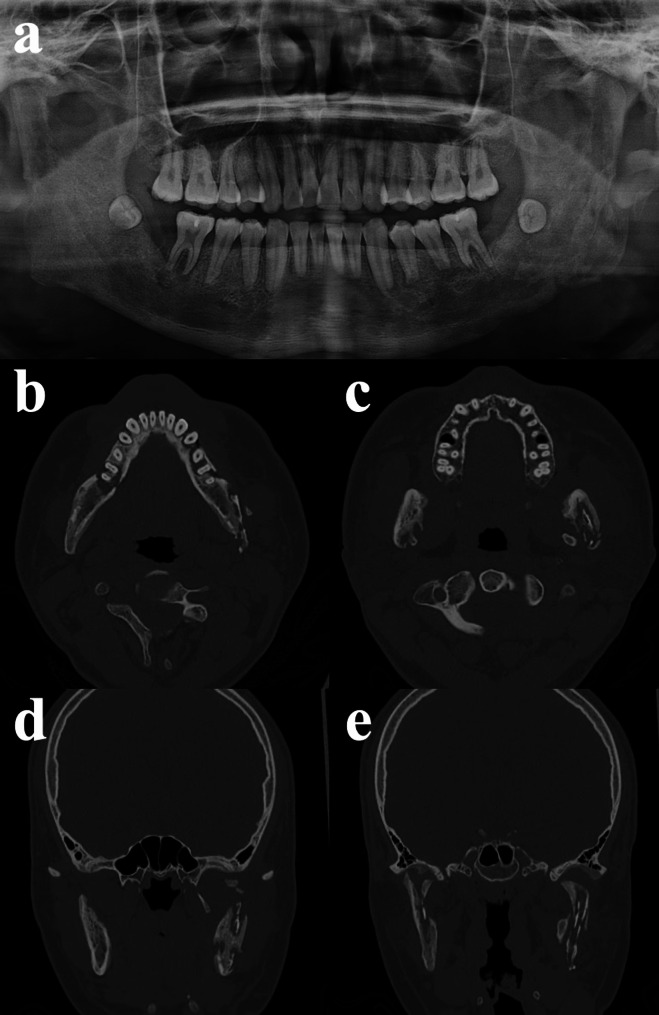
Two-month follow-up after discharge. **a** Panoramic radiograph. **b**,** c** Axial CT images. **d**,** e** Coronal CT images

At the two-month follow-up, radiographic images demonstrated diffuse chronic osteomyelitic changes throughout the mandible, including both condyles (Fig. 3). Radiolucent areas were more prominent on the left, accompanied by widening of periodontal ligament spaces and irregular bone resorption around multiple teeth. Intraoral examination revealed erythema and alveolar bone exposure along the buccal gingiva of the posterior segments. The patient remained asymptomatic, and mouth opening progressively improved to three finger breadths (37 mm). Three months after discharge, he was diagnosed with chronic multifocal osteomyelitis and commenced on antibiotic therapy combined with piroxicam.

**Fig. 4:**
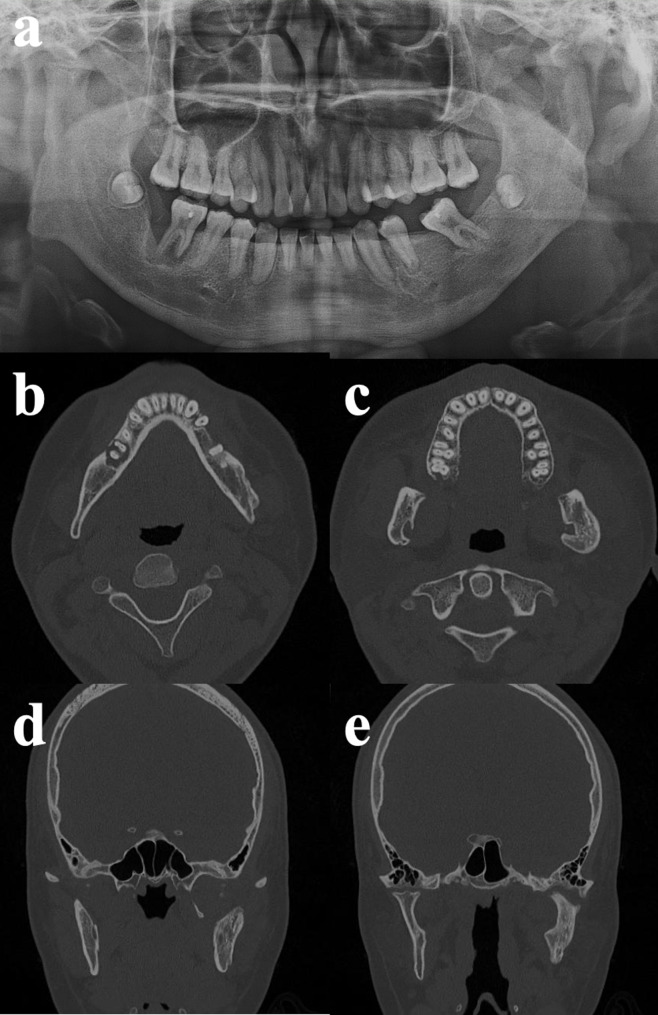
Nine-month follow-up after discharge. **a** Panoramic radiograph. **b**,** c** Axial CT images. **d**,** e** Coronal CT images

At four months, panoramic imaging showed progression of osteolytic changes involving the left mandibular condyle, ramus, and angle, with partial sequestrum formation. The left mandibular second premolar was extracted due to persistent pain. At five months, imaging revealed mixed osteolytic and sclerotic patterns consistent with the intermediate stage of osteomyelitis. By seven months, osteolysis had stabilized and progressive sclerosis was observed. No further radiographic progression was noted at nine months (Fig. 4). However, both condyles and rami exhibited deformity, and pronounced alveolar bone loss was present around the right mandibular first molar. Final follow-up CT demonstrated mixed osteolytic and sclerotic changes in both condyles, more severe on the left side (Fig. 4b, c,d, e). The patient’s maximal mouth opening ultimately recovered to 44 mm.

## Conclusion

The pathophysiology of LS is generally understood as a stepwise progression from a localized oral or oropharyngeal infection to systemic septic complications. *Fusobacterium necrophorum*, the primary etiologic agent is known to produce potent endotoxins and to promote platelet aggregation, thereby facilitating thrombus formation [11]. Following disruption of the oral or oropharyngeal mucosal barrier, the organism may invade the deep neck spaces and extend to adjacent venous structures. Previous literature has demonstrated that odontogenic LS may be accompanied by serious sequelae such as septic pulmonary embolism and deep neck infections [10]. Thus, LS often begins as a seemingly benign oropharyngeal or oral infection but can rapidly progress to severe complications if not promptly recognized.

Osteomyelitis is one of the recognized complications associated with LS, and several reports have illustrated the relationship between Fusobacterium species—the primary etiologic agent of LS—and osteomyelitis [12, 13]. Although osteomyelitis involving various anatomical sites has been described, only two published cases have reported mandibular involvement [8, 9]. In both cases, acute osteomyelitis preceded the onset of LS, which aligns with the typical presentation pattern observed in previously documented LS-associated osteomyelitis.

In contrast, the present case is distinguished by the development of chronic mandibular osteomyelitis after the diagnosis and treatment of LS, a presentation that appears to be exceedingly rare. This case demonstrates the progressive radiographic evolution of chronic osteomyelitis, including extensive osteolysis, subsequent sclerosis, and ultimately deformity of both mandibular condyles. The sclerotic changes observed on follow-up CT scans are characteristic of chronic osteomyelitis and suggest that the initial septic insult associated with LS may contribute to compromised local host defenses, allowing the infection to spread to the mandible.

Only three previously published cases have documented delayed-onset osteomyelitis occurring during post-LS follow-up (Table 2). Table 2 reveals several consistent clinical patterns among patients who developed osteomyelitis following LS. Notably, all delayed-onset cases exhibit a chronic osteomyelitis pattern. Also in all cases, prolonged antibiotic therapy was implemented, resulting in stabilization of osteomyelitic progression. These findings emphasize the importance of diligent follow-up in LS patients and suggest that extended, conservative antimicrobial management may be warranted when chronic osteomyelitis emerges as a delayed complication.

Combined LS and osteomyelitis can lead to significant morbidity including chronic pain and functional impairment [14], pathologic fracture [15], and irreversible structural deformity, as demonstrated in the present report. Therefore, early diagnosis and timely initiation of treatment are crucial. Antibiotic therapy remains the cornerstone and most effective initial approach for LS management [16]. Although the optimal duration of antibiotic administration has not been firmly established, reported regimens vary widely—from 10 days to 20 weeks—depending on clinical severity and the degree of septic involvement. A general recommendation of 2 to 6 weeks is commonly cited [17].

**Table 2:** Reported cases of osteomyelitis occurring during follow-up after initial diagnosis of LS

Author (Year)	Age/Sex	Management of LS	Site of osteomyelitis	Type of osteomyelitis	Management of osteomyelitis	Sequelae
Kempen et al. (2015) [14]	27/M	Antibiotics	Multilevel of vertebra	Chronic	Antibiotics	Persisting back pain Limited range of motion
Vogt et al. (2017) [15]	16/F	Antibiotics Anticoagulation	Right femur	Chronic	Antibiotics	Pathological fracture of right femoral neck
Latif et al. (2021) [18]	20/M	Antibiotics Surgical procedure	Right femur	Chronic	Antibiotics	Recovered
Present case (2025)	16/M	Antibiotics Surgical procedure	Mandible	Chronic	Antibiotics	Bilateral condylar deformity

The use of anticoagulation as part of initial LS management remains controversial. Because thrombosis in LS is primarily driven by infection, several authors have argued that adequate antimicrobial therapy alone is sufficient for thrombus resolution, without routine use of anticoagulation [19]. This perspective is supported by studies demonstrating no significant differences in clinical outcomes between anticoagulated and non-anticoagulated patients who received appropriate antibiotic therapy [20, 21].

Nevertheless, anticoagulation may be considered in selected circumstances, including cases with poor response to antibiotics alone [22, 23], underlying thrombophilia [24], or extensive thrombosis with risk of intracranial extension or distant embolization [25]. In this context, anticoagulation should be regarded as an adjunctive therapy reserved for carefully selected patients rather than a routine component of LS management.

Although antibiotic therapy—with or without anticoagulation—is the primary treatment modality for LS, surgical intervention may be necessary under certain conditions. If a patient fails to respond adequately to conservative medical therapy or develops persistent or worsening septic thrombosis or severe sepsis, surgical management must be considered [26]. IJV ligation or excision, once more frequently employed, is now rarely indicated. Contemporary reports suggest that such procedures should be reserved only for exceptional cases of refractory infection not responding to conventional therapy [10]. Thus, surgery should be regarded as a secondary but effective option for cases in which antibiotic therapy alone proves insufficient. As illustrated by the present case, early interdisciplinary collaboration is essential for timely surgical decision-making in LS patients.

This case also represents an unusual presentation involving thrombophlebitis of the EJV and facial vein. Given that LS classically affects the IJV, this pattern is considered atypical [1]. Anatomically, venous drainage from the oral and oropharyngeal regions most commonly flows into the IJV. Therefore, LS is generally not associated with the EJV unless an anatomic variation is present [27]. However, aberrant venous connections between the oropharyngeal venous plexus and the EJV have been reported, and such variations are estimated to occur in approximately 9% of the population [28].

This case highlights delayed-onset chronic mandibular osteomyelitis as a rare but clinically important sequela of LS. Unlike previously reported cases in which osteomyelitis preceded LS, mandibular involvement in the present case developed during post treatment follow-up, underscoring the need for long term surveillance even after apparent clinical resolution. Awareness of this atypical course may facilitate earlier recognition and appropriate management, particularly in dental and maxillofacial practice.

## Supplementary Information

Supplementary Material 1.

## Data Availability

No datasets were generated or analysed during the current study.

## Abbreviations

LS: Lemierre’s Syndrome
IJV: Internal Jugular Vein
CT: Computed Tomography
MRI: Magnetic Resonance Imaging
EJV: External Jugular Vein
VATS: Video-Assisted Thoracoscopic Surgery
